# A Fuzzy Integral Ensemble Method in Visual P300 Brain-Computer Interface

**DOI:** 10.1155/2016/9845980

**Published:** 2015-12-24

**Authors:** Francesco Cavrini, Luigi Bianchi, Lucia Rita Quitadamo, Giovanni Saggio

**Affiliations:** ^1^Department of Computer, Control and Management Engineering, University of Rome “La Sapienza”, 00185 Rome, Italy; ^2^Department of Civil Engineering and Computer Science Engineering, University of Rome “Tor Vergata”, 00133 Rome, Italy; ^3^Department of Electronic Engineering, University of Rome “Tor Vergata”, 00133 Rome, Italy

## Abstract

We evaluate the possibility of application of combination of classifiers using fuzzy measures and integrals to Brain-Computer Interface (BCI) based on electroencephalography. In particular, we present an ensemble method that can be applied to a variety of systems and evaluate it in the context of a visual P300-based BCI. Offline analysis of data relative to 5 subjects lets us argue that the proposed classification strategy is suitable for BCI. Indeed, the achieved performance is significantly greater than the average of the base classifiers and, broadly speaking, similar to that of the best one. Thus the proposed methodology allows realizing systems that can be used by different subjects without the need for a preliminary configuration phase in which the best classifier for each user has to be identified. Moreover, the ensemble is often capable of detecting uncertain situations and turning them from misclassifications into abstentions, thereby improving the level of safety in BCI for environmental or device control.

## 1. Introduction

The last two decades have seen an increasing interest in Brain-Computer Interface (BCI) as a means of communication and control for patients affected by severe neuromuscular disorders. A BCI can be regarded as a direct communication channel between a user's brain and a device; it does not rely on the conventional neuromuscular output pathways and it is therefore suitable for those suffering from the* locked-in syndrome* [1]. Nowadays, one of the leading directions in BCI research is concerned with the development of noninvasive systems in which the subject's brain activity is measured through electroencephalography (EEG). This is because those BCIs are relatively inexpensive and, as EEG recording does not require strict environmental conditions, such as those needed by functional Magnetic Resonance Imaging and Magnetoencephalography, promising for real use outside of research laboratories [1].

**Figure 1:**
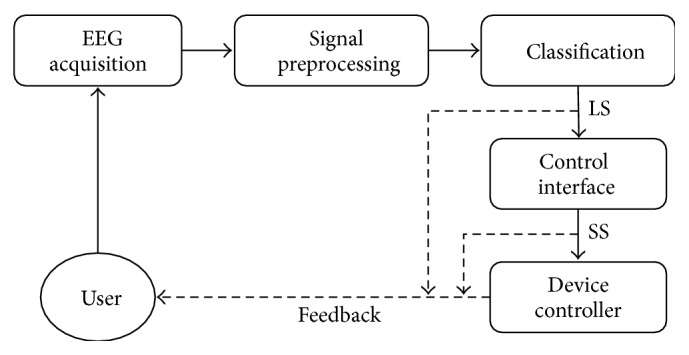
Logical schema of a BCI. After acquisition, signal preprocessing occurs and then one Logical Symbol (LS) is produced by means of a classifier. One or more LSs are then translated into a Semantic Symbol (SS) which is used for device control. The user may receive feedback from the system in one or more of the different stages.

Figure 1 depicts the logical schema of a generic BCI. The translation of user's intents into commands towards an external peripheral is a complex multistage process in which pattern recognition holds a fundamental role. For each feature vector **x** in input, the classification phase outputs the Logical Symbol (LS) that **x** is expected to encode, that is, the class to which **x** is expected to belong. Logical symbols may not have any semantic meaning; it is the Control Interface that transforms one or more LSs into a Semantic Symbol (SS), which is used to control a device.

Given the importance of classification, many of the most popular pattern recognition techniques have been applied and evaluated within the context of EEG-based BCI (see [2] for a review), for example, Fisher's Linear Discriminant Analysis (FLDA), Artificial Neural Networks (ANNs), Support Vector Machines (SVMs), and Bayesian Linear Discriminant Analysis (BLDA). With respect to ensemble methods, Lee and Choi [3] applied* stacking* [4] to motor imagery recognition. Two* boosting* [5] approaches were evaluated in [6, 7], with different outcomes. Hoffmann et al. [6] reported positive results with* gradient boosting* in visual P300 BCI, whereas Boostani and Moradi [7] found that a linear classifier often outperforms* Adaboost* in motor imagery recognition. The strategy that obtained the best performance for the second dataset of the BCI Competition III was grounded on an ensemble of SVMs having their output averaged [8]. An analogous attempt was made by Johnson and Krusienski [9] with Stepwise Linear Discriminant Analysis as classifier for the P300 speller. In such a context, also an ensemble of FLDAs was considered [10]. In [11] a Multiple Classifier System (MCS) has been applied to a self-paced BCI, achieving promising results. In addition, some studies [12, 13] compared different ensemble methods and traditional EEG-based BCI classification techniques.

In this paper we focus on combination of classifiers using fuzzy integrals [14, 15], a technique that has been successfully applied in pattern recognition since the nineties but that has drawn, to the best of our knowledge, minimal interest in the BCI community. Traditionally, MCSs have been viewed as a means for improving classification accuracy and reducing its variance, and as such they have been so far, mainly, applied to BCI. In this paper, we propose the use of classifier combination in a somewhat different fashion. Our study is concerned with the development of a framework for combination of classifiers that can be applied to a variety of BCI systems with minimal effort and changes to their structure. Our investigation has been motivated by two typical EEG-based BCI issues. Firstly, it has been observed (see, e.g., [13]) that, given a BCI protocol, there is often no evidence of a single classifier outperforming all the others for all the users of the system. Thus, the use of multiple pattern recognition algorithms and the automatic, subject-specific selection of those that perform best would be a step towards the realization of BCIs ready to be used by different subjects. Secondly, in many BCI systems, misclassification has a high impact and therefore, in vague situations, abstention is valuable [16, 17]. As it integrates decisions from different sources, combination of classifiers is promising of being better at uncertainty identification than a single pattern recognition technique. It is worthy to note that such an improvement would not only affect system performance in terms of accuracy but also in usability and safety, especially in the case of BCIs for environmental or device control. To evaluate the effectiveness of the proposed approach we applied it to a visual P300 BCI system.

The rest of this paper is organized as follows. In Section 2.1 we introduce the basic principles and the structure of a generic classifier combination system.  Section 2.2 is devoted to the presentation of the theoretical concepts on which the proposed classification strategy is grounded. In Sections 2.3 and 2.4, respectively, the proposed framework and its application to visual P300 BCI are illustrated. The results obtained in the offline analysis of data from 5 healthy subjects are presented in Section 3. In Section 4 we discuss experimental findings and possible applications of the proposed approach. Finally (Section 5), we conclude and remark on future work.

## 2. Materials and Methods

### 2.1. Fundamentals of Combination of Classifiers

**Figure 2:**
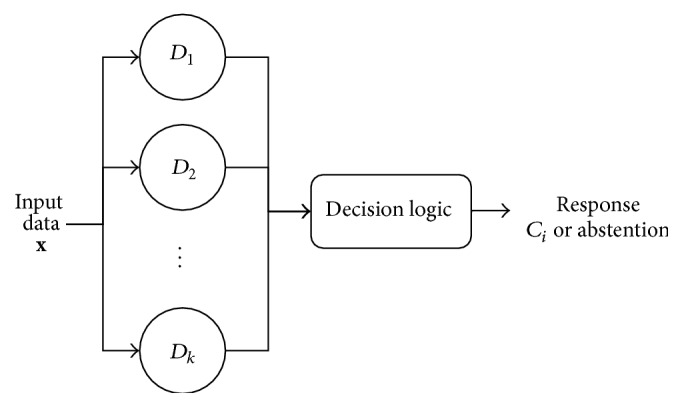
Logical schema of a classifier combination system. The input vector is classified by an ensemble of pattern recognition techniques in parallel and, from their outputs and according to its decision logic, the system returns the class to which the input vector is expected to belong or possibly abstains from making a decision.

Let *C*_1_, *C*_2_,…, *C*_*n*_ denote the possible output classes of a given pattern recognition task, and let *D*_1_, *D*_2_,…, *D*_*k*_ be *k* classifiers for that task. We will often refer to the *D*_*j*_ (*j* = 1,2,…, *k*) as* first level classifiers*. Indicating with **x** an input feature vector, with the term* combination of classifiers system* (Figure 2), we denote an ensemble method that chooses the class *C*_*i*_ to which **x** is expected to belong on the basis of the output of the classifiers *D*_1_, *D*_2_,…, *D*_*k*_ *only*. In addition, the system has the possibility of abstaining in case some predefined decision-reliability criteria are not met.

The type of combination that can be performed depends on the information provided by the first level classifiers. Drawing on the concepts introduced in [18], it is possible to distinguish tree levels in the output of a classifier:

- *Abstract level*: the classifier outputs only the class to which it assigns the input vector.
- *Rank level*: the classifier ranks all the classes in a queue at whose top is placed the most probable class for the input vector to belong to.
- *Measurement level*: the classifier assigns to each class a value representing the degree to which the input vector is believed to belong to that class.

If only information at the* abstract level* is provided, then the combination decision logic typically reduces to* voting*, whereas if the output of the first level classifiers is at the* measurement level*, more rich techniques, such as* weighted averaging* or* fuzzy integrals*, can be applied. In order for the combination to be successful, the first level classifiers should be* different* [19]. Here we use the term* different* in an informal fashion and say that two classifiers are such if (i) they are based on different algorithms (e.g., one is a feed-forward ANN and the other is based on LDA) and/or (ii) they operate on different sets of features and/or (iii) they are trained on different subsets of the available data. More formal definitions and measures of diversity in classifier ensembles lie beyond the scope of this paper and the interested reader is referred to [19].

### 2.2. Theoretical Background

We use the following notation and conventions: *∅* denotes the empty set; |*X*| and *𝒫*(*X*) indicate, respectively, the cardinality and the power set of a given set *X*; 0! = 1, as usual.

#### 2.2.1. Fuzzy Measure and Integral

Consider a decision system with *n* inputs *x*_1_, *x*_2_,…, *x*_*n*_. To express the importance of each input and of each possible coalition of inputs, we can define a measure on *X* = {*x*_1_, *x*_2_,…, *x*_*n*_}. However, in many applications, the information sources manifest some sort of positive/negative synergy when considered together, and therefore the additive property of the measure may result too restrictive. To overcome this limitation, Sugeno introduced the concept of* fuzzy measure* [20].

**Definition 1 .**

Let *X* be a finite set. A* fuzzy measure μ* on *X* is a set function defined on *𝒫*(*X*) satisfying the axioms:

(1) *μ*(*∅*) = 0.
(2) ∀*A*, *B* ∈ *X* : *A*⊆*B*⇒*μ*(*A*) ≤ *μ*(*B*).

If in addition *μ*(*X*) = 1, then the fuzzy measure is said to be* normalized*.

To aggregate information coming from the set of inputs on which we have defined a fuzzy measure, we need an extension of the (classical) integral operator, that is, we need a* fuzzy integral*. In the literature it is possible to find various definitions of integral operators with respect to fuzzy measures [21]. In this paper we will concentrate on the* Choquet integral* [21, 22]. This choice is motivated by both a theoretical property, that is, it is a proper generalization of the classical integral operator, and a practical one, that is, our learning task can be expressed as a* convex quadratic program* and therefore solved by means of well-known algorithms.

**Definition 2 .**

Let *X* be a finite set of *n* elements and let *μ* be a fuzzy measure on *X*. Let *f* : *X* → *ℛ* ^+^. Permute the elements of *X* so that 0 ≤ *f*(*x*_1_) ≤ *f*(*x*_2_) ≤ ⋯≤*f*(*x*_*n*_), where *x*_1_ denotes the first element of *X* permuted, *x*_2_ the second, and so on. The* Choquet integral* of *f* with respect to *μ*, (*𝒞*)∫*f*(*x*)*dμ*(*x*), is defined as

(1) $$\begin{array}{c} \left(\;C\;\right)\int f\left(\;x\;\right)d\mu \left(\;x\;\right)=\overset{n}{\underset{i=1}{\sum }}\left(\;f\left(\;x_{i}\;\right)-f\left(\;x_{i-1}\;\right)\;\right)\cdot \mu \left(\;A_{i}\;\right), \end{array}$$

where *f*(*x*_0_) = 0 and *A*_*i*_ = {*x* ∈ *X*∣*f*(*x*) ≥ *f*(*x*_*i*_)}.

To define a fuzzy measure on a set *X* of *n* elements, 2^*n*^ − 1 (2^*n*^ − 2 in the case of a normalized fuzzy measure) coefficients are needed. This exponential complexity is rather prohibitive and therefore, in the aim to combine the high descriptive power of fuzzy measures with the simplicity of traditional measures, Grabisch introduced the concept of *k*-*additive fuzzy measure* [23]. A *k*-additive fuzzy measure on a set of *n* elements requires $\sum_{i=1}^{k}\left(\begin{array}{c} n\\ i \end{array}\right)$ coefficients, thus being a good tradeoff between expressiveness and computational tractability.

#### 2.2.2. Importance and Interaction Index

Given a fuzzy measure *μ* on the finite set *X* = {*x*_1_, *x*_2_,…, *x*_*n*_} of system inputs, the* Shapley value* [24] can be used to estimate the contribution that each *x*_*i*_ (*i* = 1,2,…, *n*) brings to the task at hand.

**Definition 3 .**

Let *X* = {*x*_1_, *x*_2_,…, *x*_*n*_} be a finite set and let *μ* be a fuzzy measure on *X*. The* Shapley value*, or* importance index*, *v*_*x*_*i*__ of element *x*_*i*_ with respect to *μ* is defined as

(2) $$\begin{array}{c} v_{x_{i}}=\underset{A\subseteq X\setminus \left\{x_{i}\right\}}{\sum }\frac{\left(n-\left|A\right|-1\right)!\left|A\right|!}{n!}\Delta_{x_{i}}\left(\;A\;\right), \end{array}$$

where Δ_*x*_*i*__(*A*) = *μ*(*A* ∪ {*x*_*i*_}) − *μ*(*A*).

Similarly, to estimate contribution of a coalition of inputs, we can use the* extended interaction index* proposed by Grabisch [23].

**Definition 4 .**

Let *X* = {*x*_1_, *x*_2_,…, *x*_*n*_} be a finite set and let *μ* be a fuzzy measure on *X*. The* extended*, or* generalized*,* interaction index I*_*S*_ of the coalition *S*⊆*X* with respect to *μ* is defined as

(3) IS=∑A⊆X∖Sn−A−S!A!n−S+1!∑B⊆S−1S−B·μA∪B.

When the coalition is constituted by two elements, the extended interaction index reduces to the so-called (*pairwise*)* interaction index*, previously proposed by Murofushi and Soneda [25] to estimate how well two sources interact. Moreover, it can be shown [23] that the extended interaction index is a proper generalization of the Shapley value.

### 2.3. Proposed Framework

Here we give a succinct presentation of the proposed framework, highlighting the key aspects and omitting many technical details which have been already described in [26].

**Figure 3:**
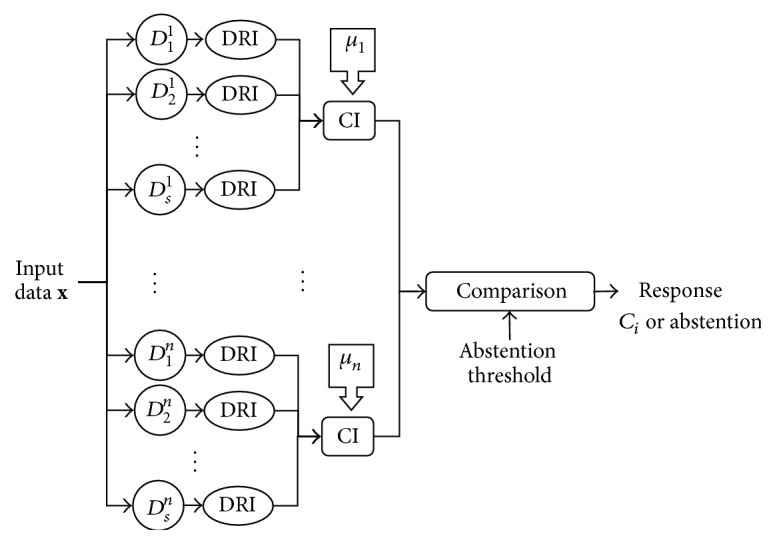
Logical schema of the proposed framework. The input vector is classified by class-specific ensembles of classifiers whose output is combined by means of the Choquet integral with respect to class-specific fuzzy measures. The system returns the class to which the input vector is expected to belong according to the combination outcomes or possibly abstains. In the figure, *D*_*j*_^*i*^ indicates the *j*th classifier in the ensemble for class *C*_*i*_; DRI indicates the mapping procedure from each classifier output space into the common, classifier-independent space; CI represents the Choquet integral with respect to the fuzzy measure *μ*_*i*_ relative to class *C*_*i*_.

Consider *k* different classifiers for the same *n*-classes pattern recognition task, and assume each classifier output is at the measurement level. Note that this assumption is not overly restrictive, as many of the most widely used algorithms (e.g., ANN) readily provide information at this level, or it is easy to extract it. For each class *C*_*i*_ we construct a logical coalition of *s* classifiers out of the *k* available; this ensemble, *E*_*i*_, includes those learners that are* best at*/*best cooperate for* recognition of input instances belonging to class *C*_*i*_. The coalition's task is providing us with some useful information about the likelihood that a given input feature vector belongs to class *C*_*i*_. Each classifier *D*_*j*_ in the coalition already computes its own likelihood value but would like to improve by combining all these scores into a global one taking into account also the worth of each learner and possible synergies among them. To this extent, we define a fuzzy measure on *μ*_*i*_ on each *E*_*i*_ and use the Choquet integral as the aggregation operator. Finally, we either output the class having the maximum likelihood or abstain if the decision seems to be too uncertain, that is, if the two top-rated classes are too close in likelihood with respect to a predefined abstention threshold.  Figure 3 shows a conceptual schema of the framework.

For the sake of clarity, in the previous paragraph we have omitted an important step. Before performing the integration we need to map each classifier output into a common and classifier-independent space; otherwise combination would not be legitimate since each learner may have its own output space (e.g., for a Bayesian classifier the output scores are typically* a posteriori* probabilities, whereas for a SVM they could be distances in the feature space). We propose the use of a simple procedure to fulfill this requirement. Firstly, linearly map the score *s*_*j*_^*i*^ assigned by classifier *D*_*j*_ to class *C*_*i*_ to a value in [−1,1] in a way such that the minimum score gets mapped to −1 and the maximum one to +1. Then, project each of those values into [0,1] by means of a sigmoid function centered between the two highest values. We can interpret the output of the procedure as the degree of belief into the proposition “*the input vector belongs to class C*_*i*_,” where 0 denotes absolute certainty that the input vector does not belong to *C*_*i*_, 1 indicates absolute certainty that the input vector belongs to *C*_*i*_, and intermediate values from 0 to 1 express monotonically increasing degrees of belief. Limiting the* slope factor* of the sigmoid function and appropriately choosing the* crossover point* (by means of a simple nonlinear optimization problem), we can also lower the scores if considerable uncertainty shines through the classifier decision; see [26] for further details.

The class-specific fuzzy measures *μ*_1_,…, *μ*_*n*_ are learned from data in the training phase [26]. The approach is grounded on least squares optimization and, due to the peculiarities of the Choquet integral, results in a convex quadratic program that can be solved using well-known and efficient algorithms.

The class-specific ensembles of classifiers *E*_1_,…, *E*_*n*_ are built according to the following principle:* a good team is made up of good players that positively collaborate towards the achievement of a common goal*. We initialize *E*_*i*_ to the empty set. Then, iteratively, we add to *E*_*i*_ the learner that best interacts with those already in the ensemble, until |*E*_*i*_| = *s*. To estimate interaction we use the extended interaction index, computed from the *s*-*additive fuzzy measure* on the entire set of classifiers. The fuzzy measure is learned from data in a preliminary training phase. Note that, since the extended interaction index is a proper generalization of the Shapley value, the first learner added to *E*_*i*_ is the most important in terms of contribution to the recognition of input instances belonging to class *C*_*i*_.

### 2.4. Application to Visual P300 BCI

This section is devoted to the application of the proposed framework within the context of visual P300 BCI. Since a vast literature (see, e.g., [1, 27–29]) already covers this BCI paradigm, we describe it briefly and instead concentrate on the issues related to the use of the proposed combination of classifiers strategy.

The most diffused protocol in visual P300-based BCI is the so-called* matrix speller*, or* P300 speller*, introduced by [27]. The subject sits in front of a computer screen that displays a 6 × 6 matrix containing alphabet letters, single digit numbers, and some commands, for example, undo and space. By making each row and column flash randomly, and asking the subject to concentrate on the item he/she wants to communicate, we induce an* oddball paradigm* which allows us to infer the desired symbol from the P300 component of the brain activity. In our experiments, each row and column flashed for 15 times; each flash lasted 100 ms and the interstimulus interval was set to 180 ms. These settings have been already used in [30]. With respect to EEG recording, we used an* EBNeuro Mizar System* (Florence, Italy), with 61 electrodes positioned according to the 10-10 international system (with reference between AFz and Fz and ground between Pz and POz), and the* NPXLab Suite* [31] for signal preprocessing and first level classification. The sampling rate was of 256 Hz, and then data was band-pass filtered between 0.5 and 30 Hz. Artifacts (e.g., eye-blinks) were removed by an expert technician. As a final remark we would like to point out that different numbers of symbols can be considered but the 6 × 6 matrix arrangement is the standard one, since it allows including all the useful characters and commands for communication. Lowering the number of symbols typically improves recognition performance but is aimed at different applications, for example, environmental control.

The first level classifiers were chosen among the most used in the P300 speller [28]: Bayesian classifier (BLDA), Artificial Neural Network (ANN), SVM with linear kernel (SVM-LIN) and with radial basis function kernel (SVM-RBF), Shrunken Regularized Linear Discriminant Analysis (SRLDA), and Stepwise Linear Discriminant analysis (SWLDA). The size of the class-specific ensembles was limited to 4 classifiers to avoid excessive computational complexity. Regarding rejection, since in the matrix speller there are no safety constraints and the objective is to maximize communication capabilities, we did not impose a fixed abstention threshold but rather chose the one that led to the best performance. In particular, we formulated this task as an optimization problem and solved it approximately using* grid search*; see [26] for further details.

## 3. Results

The experiments involved five healthy subjects (3 men and 2 women, aged from 22 to 43 years). Each experimental session was concerned with the communication of 6 different symbols. We recorded 6 sessions per subject, with a small break between two consecutive ones. Training was performed as follows. The first level classifiers learned from the first 12 symbols (2 sessions). Afterwards, the proposed framework was trained on data from the third session. Testing involved the last 18 symbols.

Concerning performance metrics, we feel that, among the ones available in the BCI field, the* efficiency* [32] is the most appropriate; this is because of the primary importance of the abstention within the proposed framework. Nevertheless, since the aforementioned index is not yet widely diffused in the BCI community, to facilitate comparison between different realizations, we report the results of the study also in terms of* Nykopp's information transfer rate* (ITR) [33].

Tables 1 and 2 report, respectively, the efficiency and the ITR of the BCI system equipped with one of the first level classifiers and the proposed framework. Experimental results support one of the main assumptions behind our investigation into combination of classifiers in BCI: even within the same protocol, there is often not a single classifier that leads to the highest performance for all the subjects. Moreover, which classifier is the best for a given user depends also on the metric being considered, for example, for subject A SVM-LIN is the best classifier according to the efficiency whereas ANN is the one with respect to the ITR. Finally, notice that for subject B the efficiency of the BCI system using ANN or SRLDA is not defined—that is, the number of misclassifications and their distribution do not allow effective communication—and that the combination performed by the framework makes the system reach a level of performance that solves the problem.

**Figure 4:**
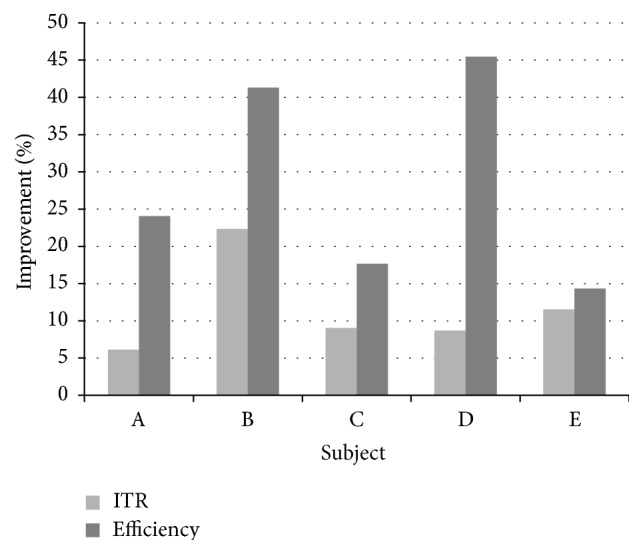
Proposed framework performance: percentage improvement with respect to the average performance of the first level classifiers.

**Figure 5:**
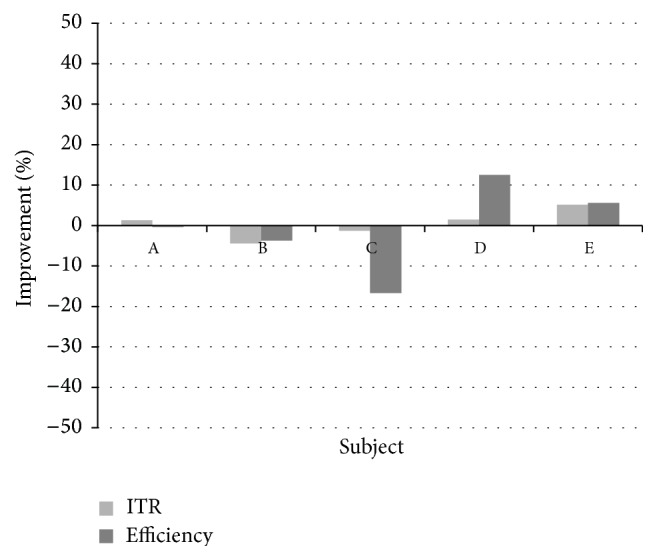
Proposed framework performance: percentage improvement with respect to the best first level classifier.

Figure 4 shows the percentage improvement, in terms both of the efficiency and of the ITR, obtained by means of the framework with respect to the average performance of the first level classifiers.  Figure 5 shows an analogous comparison with respect to the best first level classifier. Percentage improvement in efficiency ranges from more than 14% to more than 45% with regard to the average case and from less than −16% to more than 12% with respect to the best classifier. Percentage improvement in ITR ranges from about 6% to more than 22% with regard to the average case and from less than −4% to about 5% with respect to the best classifier. This makes us argue that the framework reaches a level of performance that is significantly greater than the average of the available classifiers but not necessarily higher than that of the best of them. Such a behavior is typical of multiclassifier systems [34].

**Figure 6:**
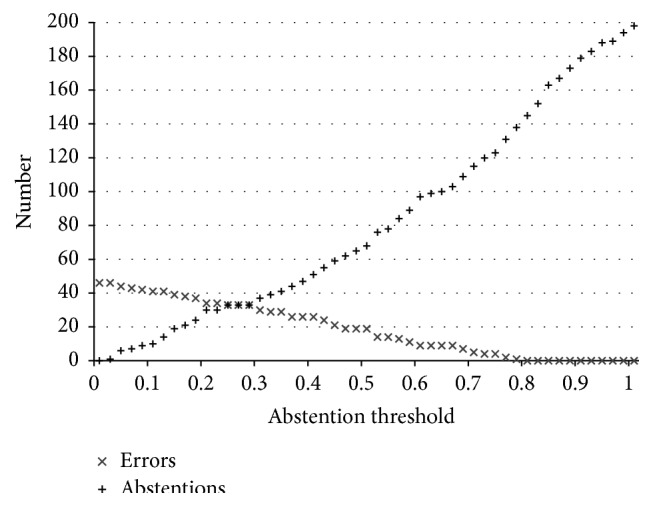
Framework error-abstention relationship as a function of the abstention threshold: subject B.

Figure 6 depicts the relationship between classification errors and abstentions as a function of the framework's abstention threshold for subject B (similar trends characterize the other users). As the abstention threshold increases, the number of errors lowers and that of abstentions rises; therefore, by combining information from multiple different sources, the framework is able to better identify uncertain situations and abstain instead of misclassifying them.

## 4. Discussion

From the experimental results and those known in the literature it is possible to argue that, even within the same BCI protocol, there is often no evidence of a single classifier outperforming all the others for all of the subjects. This leads to the need of preliminary configuration phase during which an expert has to identify the classifier that performs best for each user. The proposed framework, which for all of the subjects obtained performance higher than the average of the first level classifiers and similar to that of the best one, eliminates the aforementioned need, thus facilitating the development of systems ready to be used by different subjects.

The recognition of uncertain situations, which are often turned into abstentions instead of resulting into misclassifications, can improve the effectiveness of BCI systems for communication and the level of* safety* of those for environmental or device control. With regard to the former systems, the framework's abstention threshold can, for example, be learned in the training phase by means of the maximization of a given performance metric, whereas in the latter it can be specified* a priori* according to domain-specific constraints. Obviously, a very high threshold will avert many errors but may (and probably will) also turn some correct classifications into abstentions. A tradeoff between safety, speed, and usability has therefore to be found. In addition, the framework provides an expert/researcher with the Shapley value, which can be used for ranking the first level classifiers on the basis of their contribution to the pattern recognition process. Analogously, the interaction indexes allow the estimation of synergy among coalitions of classifiers; such a knowledge can be used, for example, to determine which pattern recognition algorithms are worthy to be considered together and which are not.

In [35], to our knowledge, the only study dealing with combination of classifiers based on fuzzy measures and integrals in the BCI literature (except for our previous studies [26, 36]), a system based on motor-imagery has been investigated and the results are reported only in terms of the error rate. A meaningful comparison of [35] and this study is therefore not possible and the only point of contact is in the MCS having a level of performance higher than the average of the first level classifiers but not always better than the best of them.

Our study concentrated on the offline analysis of data coming from healthy subjects. Two important issues are thus to be addressed: how the performance is expected to vary in the online scenario and how the system is expected to perform when used by disabled subjects. With respect to the first point, since the proposed method operates on top of the first level classifiers by aggregating their responses, we expect that possible performance differences between the offline and the online scenario will be more related to the first level classifiers behavior rather than their combination. In particular, we expect a higher variance in the base classifiers performance (e.g., due to the presence of artifacts such as eye-blinks) and therefore aggregation by means of the proposed method, which reduces classification variance, is likely to be useful and lead to results similar to those obtained in the offline analysis. With respect to the second issue, it is worthy to point out that the P300 speller has been widely and successfully used by disabled subjects [1, 29]; therefore we expect that our framework will comply with what is usually found in the literature with respect to performance with disabled users. In particular, we expect that the real advancement will be found in system usability: the proposed method, in fact, often allows avoiding misclassifications by turning them into abstentions, a fact that can surely improve system control and the effectiveness perceived by its users. This is likely to translate into users' satisfaction towards the system.

## 5. Conclusions

This study has been concerned with the application of combination of classifiers based on fuzzy measures and integrals to EEG-based BCI. In particular we concentrated on the Choquet integral and proposed a framework which can be applied to a variety of systems. To evaluate its effectiveness we have applied it to a visual P300 based BCI. Experimental testing involved the offline analysis of data relative to 5 healthy subjects. The framework performance has been compared to that of the first level classifiers in terms of both the efficiency and Nykopp's information transfer rate. Among the principal results we can cite:

(1) The best first level classifier is not the same for all of the subjects and it may also vary with the performance metric being considered.
(2) The framework obtains performance similar to that of the best first level classifier and significantly greater than their average (from more than 14% to more than 45% in terms of efficiency).
(3) The framework is often able to identify uncertain situations and turn them from misclassifications into abstentions.

Given the aforementioned results we argue that combination of classifiers using fuzzy measures and integrals is suitable for application to EEG-based BCI. The proposed framework allows realizing systems that can be used by different subjects with a good level of performance (i.e., similar to the one that could have been obtained by means of the best available classifier) without the need of a preliminary configuration phase and provides indexes for estimating the contribution that each classifier brings to the pattern recognition task and the interaction between them. The framework abstention threshold can be learnt during training or be set* a priori* according to domain-specific constraints. The latter feature is of particular importance in BCI for environmental or device control; in such systems the framework can also improve the level of safety.

For some of the subjects it has been possible to notice an increase in performance even with respect to best of the first level classifiers; this can lead to better system usability and, for example, permit lowering the number of stimuli/trials needed for the recognition of a symbol in BCI for communication. However, further investigation into such a possibility is needed. Additional future developments are the evaluation of the framework in the context of other BCI systems and, especially, its validation in online sessions.

## Figures and Tables

**Table 1:** Efficiency^a^ of the BCI system as a function of the classification technique.

Subject	BLDA	ANN	SRLDA	SWLDA	SVM-LIN	SVM-RBF	Framework
A	0.6821	0.6690	0.3277	0.3392	0.6986	0.6519	0.6964
B	0.5320	ND	ND	0.3283	0.2019	0.3874	0.5121
C	0.4194	0.2131	0.3235	0.3166	0.4559	0.2084	0.3798
D	0.4619	0.4837	0.2358	0.2774	0.5145	0.4143	0.5789
E	0.8272	0.7979	0.5948	0.7873	0.8046	0.7729	0.8736

^a^ND, which stands for not defined, indicates that the classifier performance does not allow effective communication.

**Table 2:** ITR (bit/symbol) of the BCI system as a function of the classification technique.

Subject	BLDA	ANN	SRLDA	SWLDA	SVM-LIN	SVM-RBF	Framework
A	1,8800	1,8885	1,6434	1,7259	1,8424	1,8341	1,9133
B	1,5703	1,1351	0,9654	1,2729	1,0330	1,3857	1,5010
C	1,1983	1,0374	1,0845	1,0617	1,2053	0,9610	1,1899
D	1,5542	1,4622	1,4282	1,2773	1,4989	1,4819	1,5764
E	2,1422	2,0673	1,7038	2,1059	2,0801	2,0202	2,2532
